# Epigenetics, cryptorchidism, and infertility

**DOI:** 10.1186/s12610-023-00199-7

**Published:** 2023-09-21

**Authors:** Faruk Hadziselimovic, Gilvydas Verkauskas, Michael B. Stadler

**Affiliations:** 1 Cryptorchidism Research Institute, Children’s Day Care Center, 4410 Liestal, Switzerland; 2 Children’s Surgery Centre, Faculty of Medicine, Vilnius University, 01513 Vilnius, Lithuania; 3 Friedrich Miescher Institute for Biomedical Research, Basel, Switzerland; 4 Swiss Institute of Bioinformatics, Basel, Switzerland

**Keywords:** Cryptorchidism, Infertility, Epigenetic, Chromatin remodeler, Methyltransferase, Linc RNA, GnRHa, Infertilité, Cryptorchidie, Épigénétique, ARN longs non codants, Chromatine, Méthyl transférase, GnRHa

## Abstract

**Background:**

Cryptorchid boys with defective mini-puberty and impaired differentiation of Ad spermatogonia (high infertility risk) have altered expression of several genes encoding histone methyltransferases compared to patients with intact differentiation of gonocytes into Ad spermatogonia (low infertility risk).

**Results:**

High infertility risk cryptorchid boys display hypogonadotropic hypogonadism, which, together with the diminished expression of histone deacetylases and increased expression of HDAC8 decrotonylase, indicates altered histone marks and, thus, a perturbed histone code. Curative GnRHa treatment induces normalization of histone methyltransferase, chromatin remodeling, and histone deacetylase gene expression. As a result, histone changes induce differentiation of Ad spermatogonia from their precursors and, thus, fertility. In this short report, we describe key functions of histone lysine methyltransferases, chromatin remodeling proteins, and long-noncoding RNAs, and discuss their potential roles in processes leading to infertility.

**Conclusion:**

Our findings suggest that epigenetic mechanisms are critical to better understanding the root causes underlying male infertility related to cryptorchidism and its possible transgenerational transmission.

## Résumé

**Contexte:**

Chez les garçons cryptorchides qui présentent une minipuberté défectueuse et une différenciation altérée des spermatogonies Ad (risque élevé d'infertilité), l'expression de plusieurs gènes codant pour les histone méthyltransférases est altérée par rapport aux garçons présentant une différenciation intacte des gonocytes en spermatogonie Ad (faible risque d'infertilité).

**Résultats:**

Les garçons cryptorchides à risque élevé d'infertilité présentent un hypogonadisme hypogonadotrope, qui, avec la diminution de l'expression des histone désacétylases et l’augmentation de l'expression de la décrotonyase HDAC8, indiquent une altération des marques d'histones et, par conséquent, un code d'histones perturbé. Le traitement curatif par la GnRHa induit une normalisation de l'histone-méthyltransférase, du remodelage de la chromatine et l’expression du gène de l'histone-désacétylase. En conséquence, les changements d'histones induisent la différenciation des spermatogonies Ad à partir de leurs précurseurs, et donc la fertilité. Dans le court rapport qui suit, nous décrivons les fonctions clés des histones lysine méthyltransférases, des protéines de remodelage de la chromatine et des ARN longs non codants; leur rôles potentiels dans les processus menant à l'infertilité sont discutés.

**Conclusion:**

Nos résultats suggèrent que les mécanismes épigénétiques sont un élément critique pour une meilleure compréhension des causes sous-jacentes de l'infertilité masculine liée à la cryptorchidie et sa transmission transgénérationnelle.

## Introduction

Two major goals in the field of male reproductive biology are to elucidate the molecular mechanisms that underlie cryptorchidism and to develop an effective treatment to prevent infertility. Male-specific epigenetic information involves global re-organization of and localized changes in the chromatin structure during different stages of the male germ cell differentiation [1]. Multiple studies have demonstrated that histone lysine methyltransferases regulate gene transcription, thereby influencing cell proliferation, cell differentiation, cell migration, and tissue invasion [1]. In humans, inherited alterations in nuclear maturity contributing to subfertility have been found in spermatozoids of adult males who had grandfathers diagnosed with cryptorchidism [2]. This may indicate an epigenetic mode of transmission underlying the disorder.

Using the model for artificially induced cryptorchidism, Nishio et al. found that Kdm5a (lysine demethylase 5a) expression is significantly higher in undescended testes than in descended testes. Kdm5a over-expression led to increased expression of Esr2, Neurog3, Pou5f1, Ret, and Thy1. Nishio et al. concluded that Kdm5a is likely involved in the transformation of gonocytes into spermatogonial stem cells by transcriptionally regulating specific genes via H3K4 histone modification [3].

Cryptorchidism could also be caused by prenatal exposure to external disruptors of normal embryogenesis. Any effect observed on male reproductive functions is probably due to altered epigenetic modifications following disruption of DNA methyltransferases and histone marks in the neonatal and/or adult testis [4–7].

In this short report, we present research that extends our previously published work, describe the key functions of histone lysine methyltransferases and chromatin remodeling, and summarize their role in infertility.

## Patients and methods

The patients, biopsy samples, histological analyses, and RNA sequencing protocol were described in detail in the previous study [8]. The high infertility risk (HIR) group was defined by the presence of Ad spermatogonia (< 0.005 Ad spermatogonia per tubular cross section), whereas the low infertility risk (LIR) group had a normal distribution of Ad spermatogonia [8]. Here, we interpreted the gene expression patterns observed in different prepubertal testicular cell types using our own RNA profiling data, and single-cell RNA sequencing data for adult testis (Table 1) provided by the Human Protein Atlas (www.proteinatlas.org) [9]. We analyzed the HIR and LIR groups and the HIR group before and after GnRHa treatment. Analyzed testes were not from boys with syndromic or familial cryptorchidism.

**Table 1:** Gene expression profiles in testicular cells

Gene symbol	HIR/LIR (RPKM)	log2FC/FDR	Molecular function	Testicular cell type expressing mRNA [9]
**Class 1: Leydig and/or Sertoli cells**				
*KDM6A*	17.9/16.5	0.2/0.01	Histone (lysine) demethylase	Lc/Sc
*TET1*	11.4/9.7	0.33/0.001	Functional demethylation	Lc
**Class 2: Testicular cells**				
*ARID4A*	12.5/9.0	0.48/0.03	HDAC bridging molecule	Lc/Sc/Sptg/Sptc
*ARID5B*	19.8/17.8	0.35/0.004	Histone demethylase	Lc/Sc/Sptg
*ATRX*	30.1/27.9	0.26/0.01	Methyltransferase	Lc/Sc/Sptg
*DNMT3A*	21.7/17.6	0.30/0.007	Methyltransferase	LC/Sc/Sptg
*EPC1*	20.7/17.9	0.29/0.004	Histone acetyltransferase	Lc/Sptg
*HDAC1*	18.4/17.5	n.s	Deacetylase	Lc/Sc/Sptg/Sptc
*HDAC2*	11.2/13.8	-0.28/0.03	Deacetylase	Lc/Sc/Sptg/Sptcs
*HDAC3*	17.4/17.8	n.s	Deacetylase	Lc/Sc/Sptg
*HDAC8*	7.4/6.4	0.27/0.02	Histone decrotonylase	Lc/Sc/Sptg
*INO80D*	11.1/10.3	0.24/0.02	Chromatin remodeling	Lc/Sc/Sptg
*KDM4A*	23.1/21.0	0.2/0.01	Histone demethylase	Lc/Sc/Sptg
*KMT2E*	35.6/30.7	0.29/0.01	Methyltransferase	Lc/Sc/Sptg
*PBRM1*	25.3/22.0	0.19/0.02	Chromatin remodeling	Lc/Sc/Sptg/Sptc
*PRMT2*	25.6/21.6	0.2/0.02	Arginine methyltransferase	Lc/Sc/Sptg
*SETD7*	18.7/17.7	0.22/0.047	Methyltransferase	Lc/Sc/Sptg
*SMARCA1*	81.8/78.2	0.20/0.014	Chromatin regulator	Lc/Sc/Sptg
*SMARCA2*	31.4/31.2	0.31/0.014	Chromatin regulator	Lc/Sc/Sptg
*TSPYL4*	11.8/9.4	0.32/0.01	Chromatin binding	Lc/Sc/Sptg/Sptc
**Class 3: Germ cells**				
*ARID2*	18.4/16.4	0.23/0.02	Chromatin remodeling	Sptd/Sptc
*ASH1L*	38.8/36.8	0.22/0.01	Methyltransferase	Sptg/Sptc
*BAZ2B*	42.5/38.4	0.3/0.009	Chromatin remodeling	Sptc/Sptd
*SCML2*	21.8/19.9	0.25/0.005	Transcriptional repressor	Sptg/Sptc
*SETD2*	34.8/33.2	0.19/0.02	Methyltransferase	Sptg/Sptc
*TRDMT1*	4.7/3.9	0.37/0.004	Methyltransferase	Sptc

RNA-Seq data are indicated for biopsies from high/low infertility risk patients (HIR/LIR) and samples with/without GnRH treatment. RNA levels (reads per kilobase and million, RPKM), log2 fold changes (log2FC), and false discovery rates (FDRs) are given

*n.s.* not significant, *Lc* Leydig cell, *Sc* Sertoli cell, *Sptg* spermatogonium, *Sptc* spermatocyte, *Sptd* spermatid

## Results

Different methyltransferase and chromatin remodeling genes were found to be preferentially expressed in the prepubertal testis. Relevant genes were grouped into classes based on expression in Leydig and Sertoli cells (class 1), in all testicular cells (class 2), and in germ cells (class 3; Table 1).

### Chromatin regulators show distinct testicular expression patterns

#### Class 1

*KDM6A* and *TET1* are two demethylase genes that are mostly expressed in Leydig/Sertoli cells in HIR testes (Table 1). *KDM6A* plays a critical role in the differentiation of embryonic stem cells [10]. *TET1* is predominately expressed in Leydig cells and their precursors and is highly methylated in the pluripotent state (Table 1) [11]. Its expression results in reduced cell proliferation [12].

#### Class 2

**Fig. 1:**
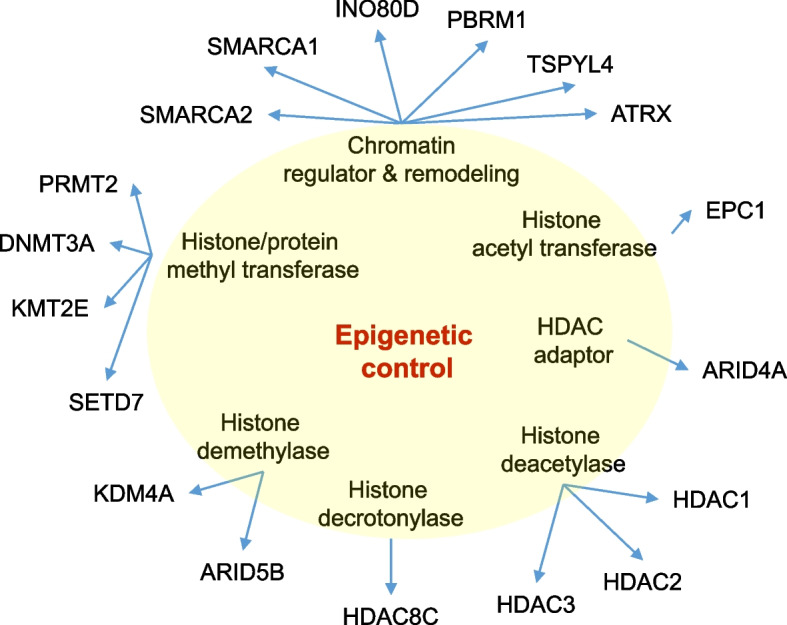
Class 2 epigenetic factors. Genes are grouped together according to their broad activities in the indicated epigenetic control mechanisms

Eighteen genes encoding modifiers of histone marks were primarily expressed in all three testicular cell types (Leydig, Sertoli, and germ cells, Fig. 1). Except for the three histone deacetylase genes, all 15 genes had increased expression in the HIR group (Table 1). ARID4A and ARID5B function as transcriptional coactivators for androgen receptor and play an integral role in androgen receptor signaling pathways [13]. Methyltransferase ATRX is an ATP-dependent chromatin remodeling factor with high homology to SWI/SNF. The protein is important for genome stability, DNA damage repair, and heterochromatin formation, and functions as a transcriptional repressor [14]. The histone acetyltransferase EPC1 is a component of the NuA4 histone acetyltransferase complex and can act as both a transcriptional activator and repressor [15]. Catalytically active methyl transferase DNMT3A is particularly active during germ cell development [16].

A putative regulatory component of the chromatin remodeling INO80D complex is involved in transcriptional regulation, DNA replication, and cell cycle control [17]. *INO80D* and *KDM4A* expression, and thus likely signaling, was enhanced in the HIR group (Table 1). Histone demethylase KDM4A participates in transcriptional repression and plays a central role in histone code modification [18]. KMT2E protein associates with chromatin regions downstream of transcriptional start sites of actively expressed genes and regulates DNA repair and apoptosis [18]. The chromatin remodeler PBRM1 acts as a negative regulator of cell proliferation, whereas the arginine methyltransferase PRMT2 is involved in histone methylation, regulation of androgen receptor signaling, and the regulation of transcription [19]. The histone lysine methyltransferase SETD7 is involved in DNA repair [20].

SMARCA genes belong to the SWI1/SNF1 family and are responsible for chromatin remodeling and DNA repair [21]. Chromatin regulators SMARCA1 and SMARCA2 act as negative regulators of chromatin remodelers by forming inactive complexes (Table 1). TSPYL4 is thought to possess chromatin and histone binding activity (see www.genecards.org for references) and is involved in cholesterol metabolism [22].

Histone deacetylases (HDACs) catalyze the removal of acetyl groups from lysine residues in histones and other proteins, often in association with transcriptional repression. We found that testicular *HDAC2* mRNA is down-regulated in the HIR group (Table 1). Expression of the decrotonylase gene *HDAC8* was increased in HIR samples (Table 1). HDAC8 protein mediates decrotonylation of histones, inducing global transcriptional regression. It acts independently, without forming any co-complexes to exert this activity [23].

#### Class 3

Six genes had increased gene expression predominately in germ cells (spermatogonia and spermatocytes, Table 1). Chromatin remodeler ARID2 is involved in transcriptional activation and repression of its target genes by chromatin remodeling, which is defined as an alteration of the DNA-nucleosome topology (see.genecards.org for reference). Methyltransferase ASH1L is required for efficient expression and H3K4 methylation of *HOXA10* [24]. Members of the BAZ2B gene family encode proteins that are integral components of chromatin remodeling complexes (see.genecards.org for reference) (Table 1). Histone transcriptional repressor SCML2 works with PRC1 and promotes *RNF2*-dependent ubiquitination of H2A, thereby marking somatic/progenitor genes on autosomes for repression [25]. SETD2 is a histone methyltransferase and represents the main enzyme generating H3K36me3, a specific mark associated with transcriptional activity that plays an essential role in the maintenance of a heterochromatic state by recruiting DNA methyltransferase DNMT3A [26]. TRDMT1 is an arginine methyltransferase, one of a group of enzymes that catalyze the transfer of methyl groups from S-adenosylmethionine to the arginine residues on histones and other proteins. This gene participates in DNA damage repair [27].

### Molecular changes following GnRHa treatment

Genes that are involved in transcriptional repression or DNA damage repair and/or negative regulation of chromatin exhibit lower expression levels after GnRHa treatment (Table 2 and 3). We found no significant differences in gene expression between the LIR group and hormone-treated HIR group (Table 2 and 3). Importantly, a previous study found no significant differences in gene expression between their LIR group and a control group [28]. Thus, lower gene expression may be interpreted as a normalization of signaling induced by testosterone stimulation during GnRHa treatment.

**Table 2:** Gene expression profiles in testicular cells before and after GnRHa treatment

Gene symbol	-/ + GnRHa treatment (RPKM)	log2FC/FDR	Molecular function	Testicular cell type expressing mRNA [9]
***Class 1***				
*KDM6A*	19.9/11.3	-0.81/0.0009	Histone demethylase	Lc/Sc
*TET1*	11.9/7.1	-0.73/0.002	Functional demethylation	Lc
***Class 2***				
*ARID4A*	12.5/9	-0.48/0.03	HDAC bridging molecule	Lc/Sc/Sptg/Sptc
*ARID5B*	21.6/15.3	-0.49/0.03	Histone demethylase	Lc/Sc/Sptg
*ATRX*	33.7/19.3	-0.79/0.002	Methyltransferase	Lc/Sc/Sptg
*DNMT3A*	24.6/19.86	n.s	Methyltransferase	LC/Sc/Sptg
*EPC1*	23.0/14.1	-0.7/0.002	Histone acetyltransferase	Lc/Sptg
*HDAC1*	22.2/12.7	-0.80/0.0008	Deacetylase	Lc/Sc/Sptg/Sptc
*HDAC2*	15.5/9.7	-0.67/0.004	Deacetylase	Lc/Sc/Sptg/Sptcs
*HDAC3*	22.2/16.2	-0.45/0.045	Deacetylase	Lc/Sc/Sptg
*HDAC8*	7.7/5.7	-0.48/0.052	Histone decrotonylase	Lc/Sc/Sptg
*INO80D*	12.1/7.9	-0.60/0.01	Chromatin remodeling	Lc/Sc/Sptg
*KDM4A*	27.6/20.3	-0.44/0.052	Histone demethylase	Lc/Sc/Sptg
*KMT2E*	35.8/24.2	-0.56/0.02	Methyltransferase	Lc/Sc/Sptg
*PBRM1*	27.2/16.6	-0.7/0.004	Chromatin remodeling	Lc/Sc/Sptg/Sptc
*PRMT2*	27.5/16.9	-0.69/0.004	Arginine methyltransferase	Lc/Sc/Sptg
*SETD7*	22.3/12.3	-0.85/0.0006	Methyl transferase	Lc/Sc/Sptg
*SMARCA1*	96.2/52.6	-0.87/0.0004	Chromatin regulator	Lc/Sc/Sptg
*SMARCA2*	37.3/24.0	-0.87/0.0009	Chromatin regulator	Lc/Sc/Sptg
*TSPYL4*	12.4/9.2	-0.43/0.054	Chromatin and histone binding	Lc/Sc/Sptg/Sptc
***Class 3***				
*ARID2*	19.3/11.6	-0.72/0.003	Chromatin remodeling	Sptd/Sptc
*ASH1L*	43.3/36.8	-0.62/0.01	Methyltransferase	Sptg/Sptc
*BAZ2B*	47.0/27.6	-0.76/0.003	Chromatin remodeling	Sptc/Sptd
*SCML2*	26.3/14.5	-0.85/0.0005	Histone transcriptional repressor	Sptg/Sptc
*SETD2*	40.0/24.5	-0.7/0.005	Methyltransferase	Sptg/Sptc
*TRDMT1*	4.89/3.5	-0.45/0.048	Methyltransferase	Sptc

RNA-Seq data are indicated for biopsies from high/low infertility risk patients before (-) and after ( +) GnRH treatment. RNA levels (reads per kilobase and million, RPKM), log2 fold changes (log2FC), and false discovery rates (FDRs) are given

*n.s.* not significant, *Lc* Leydig cell, *Sc* Sertoli cell, *Sptg* spermatogonium, *Sptc* spermatocyte, *Sptd* spermatid

**Table 3:** Gene expression profiles following GnRHa treatment in HIR samples, compared to LIR

ENTREZ ID	Symbol	Gene name	logFC	logCPM	F	*P*-value	FDR
7403	KDM6A	Lysine (K)-specific demethylase 6A	-0.5239903	6.59858773	6.38593755	0.02	0.05602502
80312	TET1	Tet methylcytosine dioxygenase 1	-0.4556459	6.53997279	5.50105813	0.03	0.0726449
5926	ARID4A	AT-rich interactive domain 4A (RBP1-like)	-0.3942475	6.06506301	5.99526693	0.02	0.06260994
84159	ARID5B	AT-rich interactive domain 5B (MRF1-like)	-0.3282121	7.22882911	3.85285411	0.06	0.12618016
546	ATRX	Alpha thalassemia/mental retardation syndrome X-linked	-0.5050769	8.25041463	6.34325962	0.02	0.05671205
1788	DNMT3A	DNA (cytosine-5-)-methyltransferase 3 alpha	-0.0408974	6.88450078	0.06435984	0.8	0.85889447
80314	EPC1	Enhancer of polycomb homolog 1 (Drosophila)	-0.4605667	6.37488169	7.09477148	0.02	0.04648734
3065	HDAC1	Histone deacetylase 1	-0.5318288	5.95514142	8.22826388	0.01	0.03546156
3066	HDAC2	Histone deacetylase 2	-0.6653504	6.56382014	12.470366	0.002	0.01557976
8841	HDAC3	Histone deacetylase 3	-0.2571251	5.35332599	2.24973919	0.15	0.23971303
55869	HDAC8	Histone deacetylase 8	-0.2757605	4.85618247	2.33455755	0.14	0.2308183
54891	INO80D	INO80 complex subunit D	-0.3696509	7.20095059	3.60611671	0.07	0.13834289
9682	KDM4A	Lysine (K)-specific demethylase 4A	-0.1773816	6.68355751	1.19093318	0.29	0.39899092
55904	KMT2E	Lysine (K)-specific methyltransferase 2E	-0.4299399	7.83891728	5.28127838	0.03	0.07790183
55193	PBRM1	Polybromo 1	-0.4968359	7.68461279	6.46367699	0.02	0.05490724
3275	PRMT2	Protein arginine methyltransferase 2	-0.4684083	7.55307237	5.8652164	0.02	0.06521626
80854	SETD7	SET domain containing (lysine methyltransferase) 7	-0.492024	7.03746884	6.5386345	0.02	0.05387161
6594	SMARCA1	SWI/SNF-related, matrix-associated, actin-dependent regulator of chromatin, subfamily a, member 1	-0.5604377	8.284278	7.53565514	0.01	0.0417173
6595	SMARCA2	SWI/SNF-related, matrix-associated, actin-dependent regulator of chromatin, subfamily a, member 2	-0.4126955	7.70083684	5.32522555	0.03	0.07678037
23270	TSPYL4	TSPY-like 4	-0.1915808	5.42985126	1.23077045	0.28	0.39031165
196528	ARID2	AT-rich interactive domain 2 (ARID, RFX-like)	-0.5031263	7.50723586	6.38425394	0.02	0.05604618
55870	ASH1L	Ash1 (absent, small, or homeotic)-like (Drosophila)	-0.4354006	8.69924223	5.4008474	0.03	0.07498705
29994	BAZ2B	Bromodomain adjacent to zinc finger domain, 2B	-0.5080609	8.74963518	6.55737462	0.02	0.053584
10389	SCML2	Sex comb on midleg-like 2 (Drosophila)	-0.4213024	6.46964321	3.87372573	0.06	0.12519553
29072	SETD2	SET domain containing 2	-0.4934595	8.21462096	6.92834399	0.02	0.04846706
1787	TRDMT1	tRNA aspartic acid methyltransferase 1	-0.2411989	5.25399927	1.48746291	0.2	0.34221114

RNA-Seq data are indicated for biopsies from high/low infertility risk (HIR/LIR) patients and samples with/without GnRH treatment. RNA levels (reads per kilobase and million, RPKM), log2 fold-changes (log2FC), and false discovery rates (FDRs) are given

*n.s.* not significant, *Lc* Leydig cell, *Sc* Sertoli cell, *Sptg* spermatogonium, *Sptc* spermatocyte, *Sptd* spermatid

### Long noncoding RNA and chromatin

Long non-coding RNAs (lncRNAs) are critical for modulating chromatin during development [29]. One lncRNA downregulated in the HIR group was *TINCR,* which produces a spliced long noncoding RNA to bind *SEDT7, ARID5B, KDM5A,* and *LINC00222*. *TINCR* is a key lncRNA required for somatic tissue differentiation, which occurs through lncRNA binding to differentiation mRNAs to ensure their expression [30]. Another lncRNA, HOX antisense intergenic RNA (*HOTAIR*), coordinates with chromatin-modifying enzymes, regulates gene silencing, and is transcriptionally induced by estradiol [31]. We found that *LINC00261* stimulates the expression of *HOTAIR* and *HOTTIP* together with *FOXA1* [8]. *HOTTIP* and *HOTAIR* expression was downregulated in the HIR group and positively responded to GnRHa treatment [8]. Similarly, *FOXA1* had decreased RNA expression in the HIR group (-1.53 log2FC; FDR 0.006) and reacted positively to GnRHa treatment (1.15 log2FC; FDR 0.03).

## Discussion

Post-translational modification of histone proteins and their interpretation by specific binding proteins, the so-called histone code, represents a fundamental regulatory mechanism that has an impact on most chromatin-templated processes, with far-reaching consequences for cell fate decisions and both normal and pathological development [32]. This epigenetic phenomenon is likely altered in HIR samples. The observed increase in gene signaling in boys with HIR may be interpreted as compensation for disturbed acetylation. GnRHa treatment induced LH and testosterone secretion, which normalizes the expression of the most methyl transferases and chromatin remodeler mRNA levels (Table 1). This is different from the results obtained with experimentally induced cryptorchidism.

In artificially induced cryptorchidism, Kdm5a overexpression led to increased stimulation of five testicular development genes [3]. In contrast, in the HIR group, developmental genes *NEUROG3, POU5F1,* and *RET* (-2.1 log2FC; 0.0001 FDR) were downregulated and *ESR2* and *THY1* were not increased compared to the LIR group [28, 33]. In addition, no differences were found in the gene expression between the LIR group and a control group [28, 33]. Therefore, the differences between the HIR group and control group would be expected to be identical to the differences in gene expression between the LIR and HIR groups. Furthermore, we could not confirm overexpression of *KDM5A*.

Of particular interest is that, in contrast to artificial cryptorchidism, GnRHa treatment led to normalization of *KDM5A* mRNA levels from 31.7 to 20.0 RPKM (-0.66 log2FC; 0.008 FDR). Thus, transformation of gonocytes into spermatogonial stem cells in humans is not a result of overexpression of Kdm5a, but involves an intact hypothalamus-pituitary–gonadal axis [28, 33]. We have described three different expression patterns of methyltransferase, deacetylase, and acetylase proteins and chromatin remodelers in prepubertal testes. It seems that chromatin formation in testicular cells requires different sets of these enzymes in different cell types.

### Estrogen effect

17-β-estradiol and its receptors are key regulators of gene transcription by binding to estrogen-responsive elements in the genome. Its receptors are important regulators of passive and active DNA demethylation [34]. Furthermore, estrogen receptor bound to estradiol recruits histone acetyl transferases (HATs), altering the balance of HATs and HDACs. The HDACs are confronted with greater amounts of acetylated histone substrates, requiring a longer time to deacetylate the acetylated histones [35, 36]. We observed increased methyltransferase gene expression and decreased deacetylase gene expression in HIR samples, indicating impaired acetylation. In pregnant women, viral infections trigger an immune response that leads to an increased concentration of 17-β-estradiol in the syncytiotrophoblast. Elevated estradiol in syncytiotrophoblasts from women who have given birth to cryptorchid boys are indicative of increased estradiol levels in the fetus [37]. Thus, hypogonadotropic hypogonadism and cryptorchidism have been hypothesized to be the result of elevated fetal estradiol levels caused by viral infection during pregnancy [37]. In a prospective study, we showed that the placentas of cryptorchid newborns had significantly higher levels of estradiol compared with control placentas of boys with bilateral descended testes [38]. Furthermore, analyzed testes were from idiopathic, non-syndromic, and non-familial cryptorchid boys who had Leydig cell atrophy, retarded tubular development implicating Sertoli cell maldevelopment, and decreased number of germ cells [38]. Testicular histology resembled that observed by Niestal et al., which was described as estrogen-induced pathological changes [39]. A negative effect of estrogen was found in the fractions of spermatozoa from infertile men with shortened ano-genital distance. This fraction is more likely to contain transposable elements harboring an estrogen receptor response element and their sperm shows substantial hypomethylation in estrogenic Alu sequences [40]. In summary, our observations are consistent with a postulated role of estrogen in modulating the expression of enzymes that modify histones, impacting the histone code.

LncRNAs have emerged as a critical layer of epigenetic regulation in which different lncRNAs are associated with distinctive epigenetic states but share a common mechanism; they physically associate with chromatin-modifying and chromatin-remodeling complexes and guide them to specific genomic loci that are crucial for proper cellular function [41]. A good example is *HOTAIR*, an lncRNA that coordinates with chromatin-modifying enzymes, regulates gene silencing, and is transcriptionally induced by estradiol [42]. *HOTAIR* expression is negatively regulated by estrogen, positively regulated by *FOXA1*, and inversely correlated with estrogen receptor expression [43].

Almost all estrogen receptor-chromatin interactions and gene expression changes are dependent on the presence of *FOXA1*. As such, *FOXA1* is a major determinant of estrogen-estrogen receptor activity [43]. Fendrr regulates Foxa1 and other genes via a Polycomb-dependent epigenetic mechanism [44]. *FENDRR* expression increased substantially following curative GnRHa treatment (2.05 log2FC; FDR 7.21E-05).

### Possible transgenerational effect of estrogen

Transgenerational epigenetic inheritance in humans has been challenged and dismissed because of difficulties ruling out the possibility that epimutation induction depends on genetic variants [45]. By generating DNA methylation-edited mice, Takahashi et al. showed that acquired methylation of CGIs can be transmitted to offspring through the parental germ line in subsequent generations of mice [45]. Moreover, they found that the CGIs with heritable DNA methylation can be demethylated in primordial germ cells. This suggests that DNA methylation memory, elicited by as yet unidentified factors, is transmitted to the next generation in mammals instead of inheritance of epigenetic information [45]. Given the commonalities in biological systems between humans and mice, Takahashi et al.’s findings may support the hypothesis that transgenerational inheritance of CGI methylation can occur in humans and, thus, could contribute to heritable susceptibility to cancer and obesity [45], as well as cryptorchidism and infertility. Reports suggest that prenatal exposure to endocrine disruptors may induce transgenerational effects on male reproductive functions, probably due to altered epigenetic modification following disruption of DNMTs and histone marks in the neonatal and/or adult testis [5, 6]. In humans, alterations of nuclear maturity able to contribute to the subfertility have been found in the spermatozoids of adult males whose grandfathers had cryptorchidism [2]. Given that histones transfer genetic material to the next generation after being transformed into protamines, abrogated histone code in HIR samples due to hypogonadotropic hypogonadism may contribute to the observed transgenerational effect in cryptorchid men. Persistent effects over several generations occur due to changes in the level of expression of master regulator genes, such as the key pluripotency gene *POU5F1*, which could contribute to propagating the epigenetic effects [46]. Pou5f1 could directly alter the expression of up to 400 genes, which in turn would modulate many downstream target genes, ultimately affecting the global transcriptional network [46, 47]. The observed *POU5F1* master regulator gene downregulation in HIR samples may contribute to propagating epigenetic effects [28].

### Can orchidopexy alone rescue fertility in high infertility risk group?

Current treatment recommendations are early orchidopexy without hormonal treatment with the expectation that successful surgery will be sufficient to protect from infertility [48–50]. However, early and successful orchidopexy fails to induce transformation of gonocytes into Ad spermatogonia in cryptorchid boys with HIR [51]. Failure to develop Ad spermatogonia results in infertility despite successful orchidopexy [51]. The incidence of HIR estimated with semi-thin sections of testicular biopsies ranges from 50 to 70% [51, 52]. Therefore, hormonal therapy provides a better chance of obtaining adequate sperm quality in adulthood [53–55].

### Limitations of the study

A critical issue, especially when working with human samples, is the number of cases that are included in a given analysis. First, the number of replicates affects the statistical confidence level. Second, human tissue samples exhibit intrinsic variability that needs to be controlled for. In this exploratory RNA profiling study, we included seven patients taken sequentially from a large ongoing study based on randomized patient samples. Their inclusion in the cohorts to be treated or to remain untreated was completely unbiased by any parameter other than undescended testes, which were surgically corrected. This sample size, though small, is sufficient for an initial transcriptome study as we present here. Furthermore, the current study lacks validation experiments for RNA profiling data. However, we previously validated the transcriptome data via qPCR [28].

In conclusion, we found impaired chromatin remodeling due to diminished expression of histone deacetylase and increased expression of methyltransferase and HDAC8 decrotonylase in HIR testes. Assuming that lncRNAs can cooperate with chromatin-modifying enzymes to promote epigenetic regulation of genes, GnRHa treatment may act as a surrogate for mini-puberty by triggering the differentiation of Ad spermatogonia via lncRNA-mediated epigenetic effects. Our observations indicate that Linc00261, *FENDRR, HOTAIR,* and *FOXA1* participate in the alternate pathway for curative GnRHa treatment to rescue impaired fertility. It is unlikely that the described epigenetic changes could be corrected by early orchidopexy. In this regard, appropriate guidance needs to be adopted for cryptorchidism treatment.

## Data Availability

Raw data files were deposited at the Database of Genotypes and Phenotypes (dbGaP) under accession number phs001275.v1.p1.

## Abbreviations

Ad: A dark spermatogonia
ARID2: AT-rich interaction domain 2
ARID4A: AT-rich interaction domain 4a
ARID5B: AT-rich interaction domain 5b
ASH1L: ASH1 like histone lysine methyltransferase
ATRX: ATRX chromatin remodeler
BAZ2B: Bromodomain adjacent to zinc finger domain 2B
CLDN3: Claudin 3
DNMT3A: DNA methyltransferase 3A
EPC1: Enhancer of polycomb homolog 1
FENDRR: FOXF1 adjacent non-coding developmental regulatory RNA
FOXA1: Forkhead box A1
GnRHa: Gonadotropin releasing hormone agonist
HDAC1: Histone deacetylase 1
HDAC2: Histone deacetylase 2
HDAC3: Histone deacetylase 3
HDAC8: Histone deacetylase 8
HOTAIR: HOX transcript antisense RNA
HOTTIP: HOXA distal transcript antisense RNA
INO80D: INO80 complex subunit D
KDM4A: Lysine demethylase 4A
KDM6A: Lysine demethylase 6A
KMT2E: Lysine methyltransferase 2E (inactive)
NEUROG3: Neurogenin 3
PBRM1: Polybromo 1
POU5F1: POU class 5 homeobox 1
PRMT2: Protein arginine methyltransferase 2
RET: Ret proto-oncogene
SCML2: Scm polycomb group protein like 2
SETD2: SET domain containing 2, histone lysine methyltransferase
SETD7: SET domain containing 7, histone lysine methyltransferase
SMARCA1: SWI/SNF-related, matrix-associated, actin-dependent regulator of chromatin, subfamily a, member 1
SMARCA2: SWI/SNF-related, matrix-associated, actin-dependent regulator of chromatin, subfamily a, member 2
TET1: Tet methylcytosine dioxygenase 1
THY1: Thy-1 cell surface antigen
TINCR: TINCR ubiquitin domain containing
TRDMT1: TRNA aspartic acid methyltransferase 1
TSPYL4: TSPY-like 4
